# A poorer nutritional status impacts quality of life in a sample population of elderly cancer patients

**DOI:** 10.1186/s12955-021-01735-7

**Published:** 2021-03-17

**Authors:** Tatiane Correia Rios, Lucivalda Pereira Magalhães de Oliveira, Maria Lúcia Varjão da Costa, Ramona Souza da Silva Baqueiro Boulhosa, Anna Karla Carneiro Roriz, Lilian Barbosa Ramos, Allain Amador Bueno

**Affiliations:** 1 Nucleo de Oncologia da Bahia (NOB), Salvador, Brazil; 2 Department of Nutrition Sciences, School of Nutrition, Federal University of Bahia, Salvador, Bahia 40110-150 Brazil; 3 Department of Nutrition, Hospital Aristides Maltez, Salvador, Brazil; 4 Food, Nutrition and Health Graduate Program, Federal University of Bahia, Salvador, Bahia 40110-907 Brazil; 5 College of Health, Life and Environmental Sciences, University of Worcester, Worcester, UK

**Keywords:** FACT-G, Cancer, Elderly, PG-SGA, Malnutrition

## Abstract

**Rationale:**

Quality of Life (QoL) is impaired in cancer, and the elderly are particularly vulnerable to malnutrition. A diagnosis of cancer in elderly patients further exacerbates risks of negative health outcomes. Here we investigated associations between QoL and nutritional status in a sample population of mostly socially deprived elderly cancer patients.

**Method:**

432 cancer patients were recruited for this cross-sectional study at point of admission to a tertiary referral hospital for cancer treatment. Patient-generated subjective global assessment (PG-SGA) assessed nutritional status. Functional assessment of cancer therapy- general (FACT-G) quantified QoL. Relationship between PG-SGA and QoL was assessed by Spearman correlation. PG-SGA outcomes were compared against FACT-G scores employing Mann–Whitney test. Bivariate Linear Regression Model was employed to investigate influences of sociodemographic, clinical and nutritional status upon QoL.

**Results:**

37.5% of participants were malnourished or at risk. 39% were illiterate and 54.6% had family income lower than minimum wage. Malnourished patients showed lower FACT-G scores (76.8 vs. 84.7; *p* = 0.000). Poor nutritional diagnosis was inversely correlated with all QoL domains. Bivariate regression analysis showed that lower PG-SGA scores (βo =  − 1.00; *p* = 0.000) contributed to FACT-G score deterioration, the male gender showed better QoL scores, and other clinical and sociodemographic variables did not show relationship.

**Conclusion:**

Poorer nutritional status was significantly associated with worsened physical, social, emotional and functional well-being QoL domains in elderly cancer patients. Poorer nutritional status is an independent risk factor for worsened QoL. Future policies aimed at particularly vulnerable populations may improve QoL and health outcomes.

## Introduction

Increased life expectancy is positively associated with increased incidence of cancer. Approximately 60% of new cancer cases are diagnosed in the population group aged 65 years or older, and approximately 70% of the mortality attributed to cancer occurs in that group [1]. Ageing is positively associated with physiological and functional deterioration, which are important risk factors for the exacerbation of chronic conditions [2]. Decline of functionality is also associated with increased morbidity, higher hospitalization rates and increased costs of healthcare provision [3].

The provision of nutritional advice for patients with cancer must be tailored to the patient’s specific needs. Particular attention is required for elderly cancer patients when considering their naturally expected physiological and functional decline. Weakening attributed to loss of muscle mass and reduced movement is expected in ageing; however, when associated with a diagnosis of cancer, the sufferer’s quality of life (QoL) and life expectancy become dramatically compromised [4].

The impact of cancer and chronic conditions in the social and emotional aspects of the sufferer’s life is significant, particularly in the elderly. The need for lifestyle restructuring, and the sometimes-dramatic changes in lifelong-acquired habits, may negatively influence their mental health and personal values, as well as their social structure [5]. By identifying deficits in QoL in cancer sufferers, adjustments can be made to promote humanization of the care provided to those patients [6].

Nutritional support is fundamental in therapies for cancer patients, and even more important for those with long-term limited access to healthcare. Different methods of nutritional diagnostics are consistent in revealing associations between poor nutritional conditions with worsened outcomes in post-surgical complications, survival, and mortality. A recent meta-analysis investigating malnutrition in elderly cancer patients showed that, amongst other results, food intake reduction was associated with increased risk of mortality (OR: 2.15; 95% CI 1.61–2.86), that lowered prognostic nutritional index (PNI) scores in the pre-surgery period were associated with lowered survival (HR: 1.89; 95% CI 1.03–3.48), and that low geriatric nutritional risk index (GNRI) scores were associated with increased risk of post-surgery complications (HR: 2.02; CI 1.13–3.66). In the opposite direction, normal GNRI (≥ 98) was associated with improved survival (HR: 1.672; CI 1.079–2.581) [7].

Large scale populational studies investigating malnutrition in elderly cancer patients in Brazil are scarce. A multicentre study carried out in Brazilian hospitals across the country identified malnutrition in 48.1% of patients. However, its prevalence was not homogeneous nationwide, and was much more prevalent in the North and Northeast regions of Brazil, where the per capita income is lower as compared to the South and Southeast regions [8]. Furthermore, that study found that malnutrition was correlated with the primary diagnosis at admission, age (60 years old and older), presence of cancer or infection, and longer hospital stay [8]. A survey investigating the prevalence of hospital malnutrition in the elderly found that it varied from 20 to 60.6% in different regions across Brazil, but which was also dependent upon the diagnostic methods employed [9].

A more thorough understanding of the associations between nutritional status and QoL in elderly cancer patients may contribute to the further development of strategies that support better health outcomes for sufferers. The aim of the present study was to investigate potential associations between nutrition status and parameters of QoL in a sample population of mostly socially deprived elderly patients with a diagnosis of cancer.

## Method

This cross-sectional study received full ethical approval. The study location was a charitable hospital, reference centre for tertiary medicine in the state of Bahia, Brazil. All procedures were conducted in full compliance with the ethical standards of the research committees associated with the study and with the 1964 Helsinki Declaration and its subsequent amendments.

All patients were explained the aims and objectives of the study, and those who agreed to participate signed the ‘Free and Informed Consent Form’, which included a clause on ‘Consent to Publish’. No participant can be identified in this study. Patients with an associated psychiatric diagnosis, as well as patients admitted to hospital for the sole purpose of diagnostic investigations, were not included. Inclusion criteria included individuals aged 60 years or older, admitted to medical and surgical wards of the hospital for cancer treatment. All patients had their cancer diagnosis confirmed prior to participation in this study.

Between June 2013 and January 2014, 461 consenting patients were recruited, however 29 participants did not complete the assessment. The research staff in charge of data collection were Registered Dietitians with full training on the usage of the questionnaires adopted in this study, therefore minimizing the likelihood of errors in data collection. A standardized form collected demographic data (age, gender, place of residence, literacy, family income and occupation) and clinical data including type of diagnosed cancer, current treatment plan (surgery, chemotherapy, venous catheter blockage management), and comorbidities.

PG-SGA, adapted by Ottery [10], translated to the Portuguese language and validated by Gonzalez et al. [11], consists of a questionnaire divided into two sections. The first section addresses weight loss, changes in dietary intake, symptoms related to cancer, and changes in functional capacity. The second section addresses factors associated with a diagnosis of increased metabolic demand, and physical examination. PG-SGA scores range from 0 to 35, with a higher score reflecting a greater risk of malnutrition, and the three possible outcomes are ‘A’ for well-nourished patients, ‘B’ for those with suspected or moderate malnutrition, and ‘C for severely malnourished patients. For the purpose of this study specifically, we have grouped the outcomes of the PG-SGA into two categories: ‘A’ for well-nourished patients, and ‘B + C’ for at risk and malnourished patients.

PG-SGA, the tool chosen for this study, is a validated protocol for use in cancer patients 18-years old and older. It is inexpensive and relatively easy to execute, and can detect nutritional changes in its early stages, allowing for early nutritional interventions. PG-SGA correlates well with other nutritional assessment methods [12] and malnutrition identified by this method has been shown to be a good predictor of unfavourable clinical outcomes in cancer patients [13, 14].

Quality of life was measured using the functional assessment of cancer therapy: general (FACT-G), version 4 instrument, as previously described [15]. The FACT-G is a functional scale consisted of 27 items that assess four QoL domains: physical well-being (PWB) with 7 items, functional well-being (FWB) with 7 items, social/family well-being (SWB) with 7 items, and emotional well-being (EWBE) with 6 items. Answers are presented on a five-point Likert scale (0 = not at all, 4 = very much). The scores for each of the four domains are added, and the overall QoL score is also added. Higher values correspond to better QoL. The questionnaire is self-administered. The FACT-G was translated into Portuguese and validated by Pereira and Santos [16] with results that show good global internal consistency, good test–retest fidelity and good sensitivity for the study of QoL in cancer patients.

### Statistical analysis

We employed descriptive statistics to analyse the frequencies, means, standard deviation (SD) or medians, and interquartile ranges of the investigated variables for the study population. To investigate assumptions of normality, a boxplot, histogram, and the Kolmogorov–Smirnov test were used.

The Spearman’s correlation was used to identify the relationship between PG-SGA and FACT-G scores. For the interpretation of the magnitude of the correlations, the following correlation coefficient classification was adopted: 0.00–0.19 = absent or very weak correlation; 0.20–0.39 = weak correlation; 0.40–0.59 = moderate correlation; 0.60–0.79 = strong correlation; 0.80–1.0 = very strong correlation, as previously described [17].

The Mann–Whitney test was used to compare the medians of FACT-G domains with the nutritional status (PG-SGA: Well-nourished *vs.* Risk + Malnourished). The T-test was used to compare the means of the FACT-G global score with the nutritional status.

As the global FACT-G score was normally distributed, we initially performed a bivariate linear regression to examine associations between nutritional status (independent variable of interest) and QoL (dependent variable), and also to explore whether other demographic (sex, age, income, education), clinical (cancer location, presence of comorbidities such as diabetes, hypertension, liver, kidney and heart disease) or lifestyle characteristics (physical activity, alcohol intake and smoking) were possibly associated with QoL.

Amongst the covariates investigated (sociodemographic, lifestyle and clinical data), only sex showed statistical significance in the bivariate analysis. In the multivariate analysis, when testing for associations between nutritional status and sex-adjusted QoL, the statistical significance is lost. Thus, we chose to present only the bivariate analysis, as nutritional status and sex were shown to be independently associated with QoL in the study population.

*p* < 0.05 was considered statistically significant for all tests. Statistical analysis was performed using the SPSS statistical package (version 20.0, IBM) and Stata. The statistical analyses have been reviewed by a qualified statistician.

## Results

Four hundred thirty-two patients completed the study. The median age for all participants was 67 years, with the P25-P75 interquartile range (IQR) 63–74), the median age for men only was 69 (IQR 64–75), and the median age for women was 66 (IQR 63–73) (Table 1). Forty-five % of participants were males. Sixty-six % of patients lived away from Salvador and surrounding metropolitan Salvador. Self-declared illiteracy was identified in 39% of participants. The total family income of 54.6% of participants was lower than minimum wage. The most frequent occupation was farming and agriculture, in 42.2% of cases. The most prevalent type of cancer diagnosed was prostate, in 19.9% of cases, followed by skin (17.1%), cancers of the gastrointestinal tract (16%), breast (15.7%) and gynaecological tract (7.7%). Hypertension was a comorbidity in 60.2% of cases, followed by diabetes mellitus (17.1%) and other types of cardiovascular disease (10.9%). The vast majority of patients were admitted to hospital for cancer surgery (98.6%), whilst 0.9% were admitted for chemotherapy and 0.5% were admitted to manage venous catheter blockage. The PG-SGA showed that 62.5% of patients were well nourished at hospital admission, whilst 29.9% were either at risk of malnutrition or moderately malnourished, and 7.6% were severely malnourished (Table 1).

**Table 1:** Demographic and clinical characteristics of the sample population of elderly cancer patients investigated

Age, all participants (median years, IQR)	67, 63–74	
Male (median years, IQR)	69, 64–75	
Female (median years, IQR)	66, 63–73	
Sex	N	**%**
Male	196	45.0
Female	236	55.0
Place of residence		
Salvador (Capital city) and greater Salvador	147	34.0
Other regions in the state of Bahia	285	66.0
Literacy		
Illiterate	167	39.0
Literate	261	61.0
Family income		
≤ MW	231	54.6
> MW	192	45.4
Occupation		
Farmer	177	42.2
Housewife	40	9.6
Housekeeper	16	3.8
Others	186	44.4
Diagnosed cancer		
Prostate	86	19.9
Skin	74	17.1
GIT	69	16
Breast	68	15.7
Gynecological	33	7.7
Urological	31	7.2
Head and Neck	30	6.9
Bone	25	5.8
Pulmonary	12	2.8
Others	4	0.9
Comorbidities		
SAH	260	60.2
DM	74	17.1
Cardiopathy	47	10.9
Hepatopathy	23	5.3
CKD	21	4.9
Current Treatment		
Surgery	426	98.6
Chemotherapy	4	0.9
Venous catheter blockage management	2	0.5
PG-SGA		
Well nourished (A)	270	62.5
Risk or moderate malnourishment (B)	129	29.9
Severely malnourished (C)	33	7.6

*CKD* chronic renal disease, *DM* diabetes mellitus, *GIT* gastrointestinal tract, *IQR* interquartile range, *PG-SGA* patient-generated subjective global assessment, *SAH* systemic arterial hypertension

**Fig. 1:**
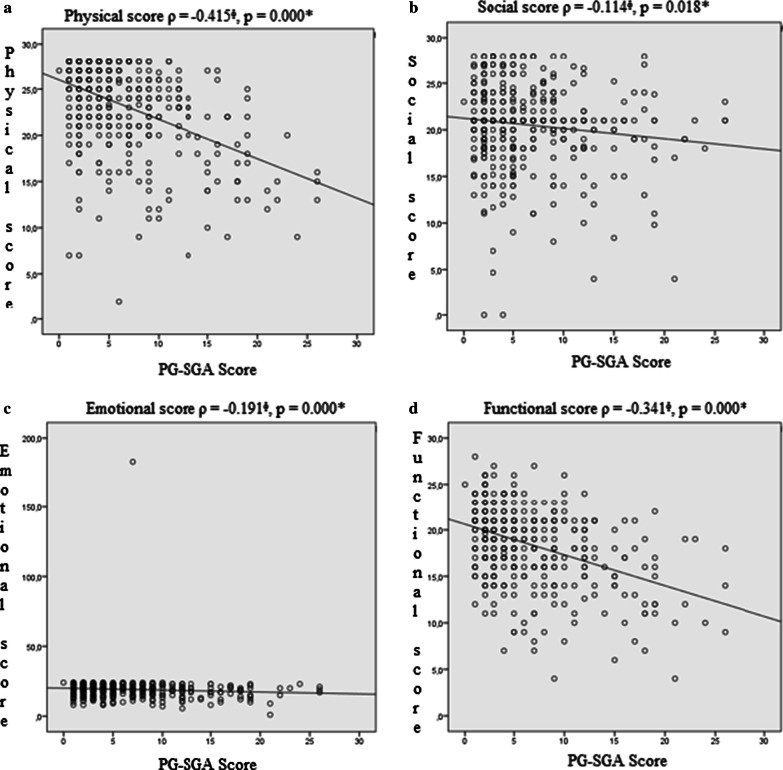
Spearman correlations between FACT-G domains scores and PG-SGA scores in the sample population of elderly cancer patients investigated. *Spearman correlation ^ϕ^*p* value < 0.05. *PG-SGA* patient-generated subjective global assessment, *FACT-G* functional assessment of cancer therapy: general

**Fig. 2:**
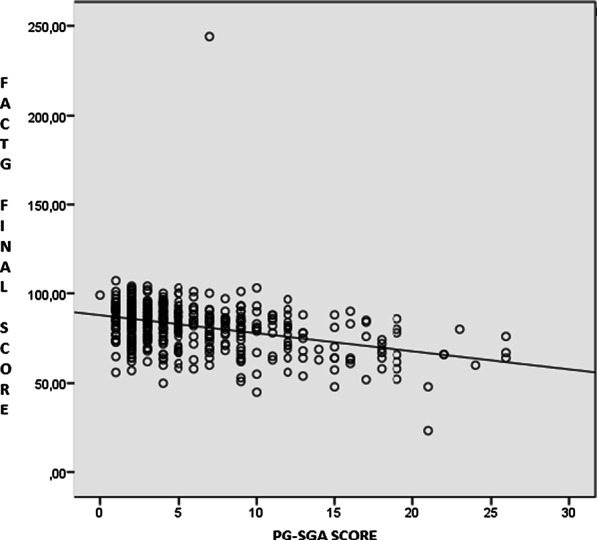
Scatterplot of the Spearman correlation between FACT-G final score and PG-SGA screening score in the sample population of elderly cancer patients investigated. Spearman correlation =  − 0.376 *p* value = 0.000. *PG-SGA* patient-generated subjective global assessment, *FACT-G* functional assessment of cancer therapy: general

Although the global FACT-G score results showed normal distribution, the FACT-G subdomains were not normally distributed. The Spearman’s correlation analysis revealed a significantly negative correlation between the four FACT-G domains (A: Physical ρ =  − 0.415, *p* = 0.000; B: Social ρ =  − 0.114, *p* = 0.018; C: Emotional ρ =  − 0.191, *p* = 0.000; D: Functional ρ =  − 0.34, *p* = 0.000) and the PG-SGA scores (Fig. 1). Figure 2 shows the significantly negative correlation between the FACT-G global score and the PG-SGA scores (ρ =  − 0.376, *p* = 0.000). Inverse correlations can be expected as low PG-SGA scores indicate better nutritional status whilst high FACT-G scores indicate better QoL.

We have found significantly higher QoL scores in the FACT-G Physical, Emotional and Functional Well-Being domains in well-nourished patients, as compared to those at risk of malnutrition or malnourished (Table 2). Only the Social and Family Well-Being QoL domain was similar between both groups.

**Table 2:** FACT-G domains median scores in relation to nutritional status, as defined by the PG-SGA, in the sample population of elderly cancer patients investigated

FACT-G domains	Well-nourished (n = 270)	Risk + Malnourished (n = 162)	*p* value*
	Median (P25-P75)^a^	Median (P25-P75)^a^	
*PG-SGA*			
PWB	26.0 (23.0–27.0)	23.0 (18.0–26.0)	0.000*
SWB	21.0 (19.4–23.0)	21.0 (18.0–23.0)	0.081
EMB	20.0 (17.0–22.0)	19.0 (15.0–21.0)	0.006*
FWB	20.0 (17.0–21.0)	18.0 (14.0–21.0)	0.000*
	Mean (SD)^b^	Mean (SD)^b^	
Final score	84.7 (0.9)	76.8 (1.0)	0.000**

*PG-SGA* patient-generated subjective global assessment, *FACT-G* functional assessment of cancer therapy: general, *PWB* physical well-being, *SWB* social and family well-being, *EMB* emotional well-being, *FWB* functional well-being

^a^P25 = 25th percentile 25, P75 = 75th percentile. ^b^SD = standard deviation. *Mann Whitney *p* value < 0.05. **T test with equal variances *p* < 0.05

The bivariate linear regression analysis amongst the sociodemographic, clinical and nutritional variables showed a statistically significant association between the QoL FACT-G scores with the nutritional status, as assessed by the PG-SGA scores, and with the male gender. Increasing PG-SGA by 1 point decreased the QoL FACT-G by 1 point, whereas the male gender added 2.88 points to the FACT-G score (Table 3).

**Table 3:** Bivariate linear regression analysis between FACT-G QoL scores with the PG-SGA-measured nutritional status and gender in the sample population of elderly cancer patients investigated

Variables	FACT-G				
	Coefficient (β_0_)	IC	*p* value	F-value	Prob > F
PG-SGA	− 1.00	− 1.25; − 0.76	0.000*	63.24	< 0.001
*Gender*					
Male	2.88	0.17; 5.59	0.037*	4.35	0.04
Female	–				

^*^Bivariate Linear Regression Model, *p* < 0.05

## Discussion

In the present study, 432 elderly patients with a diagnosis of cancer admitted to a tertiary referral charitable hospital for cancer treatment completed the PG-SGA and the FACT-G assessment tools, as previously established [10, 11, 15]. We have found a high percentage of individuals at risk of malnutrition or malnourished at point of admission, which further evidences the need for early nutritional intervention to preserve QoL and to improve health outcomes. It has been previously demonstrated that nutritional interventions in adult cancer patients have improved QoL [18].

A cohort study of elderly cancer patients undergoing chemotherapy identified low QoL scores at baseline, and a further deterioration after chemotherapy [19]. That study also showed that as the treatment progressed, other undesired effects also occurred, including deteriorated nutritional status, as identified by the Mini Nutritional Assessment tool [19]. A study recruiting 60 cancer patients aged in average 61.9 years receiving radiation therapy identified a significant correlation between changes in PG-SGA scores (*p* < 0.001) and changes in QoL scores (*p* = 0.003) amongst the patients that either improved (5% of participants), maintained (56.7%) or deteriorated (33.3%) their nutritional status after 4 weeks of treatment [14]. The researchers’ regression analysis revealed that 26% of the QoL change variation was attributed to changes in the PG-SGA scores [20].

Previous cancer studies have shown that not only malnutrition, but also nutritional risk, are associated with poorer QoL and prolonged hospital stay, irrespective of age at diagnosis and type of surgical procedure performed [21, 22]. A small sample population study of malnourished elderly patients submitted to gastrointestinal cancer surgery showed a mortality rate of 1/3 of the cohort 8 months after the surgery [23]. That study showed higher surgery-related complication rate in comparison to non-malnourished patients. The study also found that early nutritional monitoring could assist in better recovery during the postoperative period and improve QoL one year after surgery [23].

In our study, the FACT-G Physical Well-Being and Functional Well-Being domains were inversely correlated with PG-SGA scores. In the context of our sample population, specifically, we found these results extremely relevant; it is likely that the manifestations of cancer-related symptoms could be a catalyst for the lack of motivation to perform daily activities. Depending on family and social circumstances, this could be as difficult as the inability to successfully care for oneself. Our findings further corroborate the need for nutritional intervention as early as possible in the course of the disease.

Capuano et al. [24] assessed nutritional status using PG-SGA and performance status using the Eastern Cooperative Oncology Group PS tool in a sample population of 61 patients with head and neck cancer. The authors found that malnourished patients presented more frequent complaints of fatigue, weight loss, nausea and vomiting, as well as lower QoL scores, as compared to the non-malnourished group. It has been demonstrated that weight loss, reduced functional capacity, pain and fatigue are associated with shorter survival in patients with inoperable non-small cell lung cancer [25]. In a cohort study of 53 elderly cancer patient in catabolic state conducted in Sweden, a significant correlation was found between spontaneous physical activity and nutritional status, in which less spontaneous physical activity was directly correlated with greater weight loss [26].

Significant functional limitations have been reported in older breast cancer women survivors, as compared to older women without cancer [27]. Sarcopenia is a highly prevalent condition in ageing, and further exacerbated with a diagnosis of cancer, dramatically deteriorating QoL and survival rate [28–30]. The carcinogenic biochemical environment is known to induce the systemic release of pro-inflammatory cytokines and hormones associated with anorexigenic effects, including interleukin-6, interferon-y, ghrelin and leptin, as well as mediators with proteolytic actions, including Proteolysis Inducing Factor and interleukin-1 (IL-1). The effects of said molecules in tandem include decrease appetite and motivation to eat, further accentuating sarcopenia [31].

In our study, the FACT-G Emotional Well-Being domain showed a significant correlation with PG-SGA scores. Literature is still somewhat scarce in studies that have explored the relationship between the emotional life aspects of elderly cancer sufferers with their nutritional status. However, available evidence suggests that malnutrition increases by five-fold the risk of depression in elderly patients undergoing chemotherapy [32]. The occurrence of gastrointestinal symptoms and the side-effects associated with cancer treatment reduce food intake and induce weight loss, impacting mobility and the capacity to perform daily-life activities. It is also known that reduced performance and mobility have a negative impact on social interactions and emotional health of the sufferers [33]. Therefore, even though social and emotional QoL and nutritional status were found to be correlated with each other in our study, we suggest that one effect may not necessarily be a direct consequence of the other, but possibly an indirect relationship, in which one exacerbates the other via the consequences of impacted social and emotional life.

The FACT-G Social Well-Being in our study did not reach significance in the Mann–Whitney test comparing the medians of well-nourished (A) versus at risk + malnourished (B + C) groups. However, the Spearman correlation coefficient obtained from the global score showed a weak but statistically significant association. The lack of a very strong relationship between social well-being and nutritional status could be explained by the existence of family support in most of the cases.

In our study, the female sex appeared to be more negatively influenced in QoL. The bivariate linear regression analysis showed that nutritional status directly affected QoL in elderly cancer patients, but also that men were much less susceptible than women. We have not found reports in the literature that have associated nutritional status and QoL in elderly cancer male sufferers, specifically. Whilst our study included all types of cancers, the studies we have found were specific to prostate cancer, and compared different types of treatment [34, 35].

A particular aspect of our study worth of further consideration is the socioeconomical reality of our participants. In our study, nearly 40% of participants were illiterate and over 50% had a total family income below minimum wage. Most of the studies referred to in our discussion were conducted in economically developed countries. On the other hand, most of the participants in our sample population were extremely poor and socially deprived. Even though our bivariate linear regression analysis has not revealed a significant association between sociodemographic data, particularly literacy and family income, with QoL, we believe the formers could be contributing factors for the exacerbation of the latter. The ill-fated relationship between poverty and negative health outcomes has been documented several decades ago [36], and this could be one of the factors that explain why a high proportion of our participants were malnourished or at risk of malnutrition at the point of admission for cancer treatment. It is known that effective nutritional assessment followed with tailored nutritional interventions improve survival, functional status and body weight gain in elderly cancer patients [37]. Public Health policies that consider early nutritional assessment and early nutritional interventions will promote better QoL for elderly cancer patients.

This was a cross-sectional study with a non-probabilistic sample population, and our results cannot be used to propose population inference. However, we believe our sample population was a good representation of the elderly cancer population of Salvador and of the state of Bahia in Brazil, as it was recruited in a tertiary referral hospital, charitable reference centre in the state. The cross-sectional nature of our study allowed us to only report associations between nutritional status and QoL, and thus we cannot state the directionality of the relationships. In addition, without longitudinal data, our study does not provide information on how changes in nutritional status throughout the cancer trajectory may further influence QoL.

Our research findings further reinforce the relevance of early nutritional diagnosis. In addition to the well understood impact of malnutrition on post-surgical complications, survival and mortality, we have observed an association between malnutrition and worsened QoL in elderly cancer patients. Our findings reinforce the position that nutritional risk screening is necessary at the first contact with the patient, be it either as outpatient or in the hospital environment. Nonetheless, future research will identify the most effective tools that correctly identify malnutrition, cachexia and sarcopenia in elderly cancer patients. Such tool will be easily deployable in clinical practice, facilitating tailored and appropriate support.

## Conclusion

In our study, we have found that patients with better nutritional status have better functional, emotional, social and physical scores of QoL. We have also found that men were at lower risk as compared to women. Our study adds to the body of evidence that confirms the relationship between nutritional status and QoL in cancer patients. Further studies are still needed, and it is hoped that their outcomes may pave the way for betterment of health policies aimed at highly vulnerable populations.

## Abbreviations

QoL: Quality of Life
PG-SGA: Patient-generated subjective global assessment
FACT-G: Functional assessment of cancer therapy-general
